# EFFECTS OF INCENTIVE SPIROMETER ADDED TO STANDARD REHABILITATION IN PREVIOUSLY HOSPITALIZED ADULTS WITH POST-COVID-19 CONDITION: A RANDOMIZED CONTROLLED TRIAL

**DOI:** 10.2340/jrm.v58.44926

**Published:** 2026-05-05

**Authors:** Marina KLONI, Alexandros HERACLIDES, Theognosia PANTELI, Alexios KLONIS, Panagiotis RENTZIAS, Christos KARAGIANNIS

**Affiliations:** 1 Physiotherapy Department, Rehabilitation Centre EDEN, Larnaca; 2 Department of Health Sciences, European University of Cyprus, Nicosia, Cyprus; 3 Department of Anaesthesiology, The Newcastle Upon Tyne Hospitals NHS Foundation Trust, Newcastle, UK

**Keywords:** incentive spirometer, post-COVID-19 condition, dyspnoea, peak expiratory flow, dyspnoea, rehabilitation

## Abstract

**Objective:**

Post-COVID-19 condition is associated with persistent respiratory symptoms and impaired functional capacity. This study evaluated the effects of adding an incentive spirometer to standard rehabilitation in adults with post-COVID-19 condition, with peak expiratory flow, dyspnoea, and quality of life as the prespecified primary outcomes.

**Design:**

Randomized controlled trial.

**Subjects/Patients:**

Eighty-two previously hospitalized adults diagnosed with post-COVID-19 condition, were randomly assigned to an experimental group (rehabilitation plus incentive spirometer, *n* = 41) or a control group (rehabilitation alone, *n* = 41).

**Methods:**

Eight outcomes were assessed at baseline and 1 month, including peak expiratory flow, Medical Research Council dyspnoea scale, EQ-5D-5L, and functional performance tests.

**Results:**

Both groups improve at 1-month follow-up. The experimental group showed greater improvements in peak expiratory flow (between-group difference = 65.85 L/min; 95% CI: 4.35 to 127.35; p = 0.007), and dyspnoea (MRC difference = −1.08; 95% CI: −1.54 to −0.62; *p* < 0.001) compared with the control group. No significant between-group differences were observed for quality of life or functional performance measures.

**Conclusion:**

Adding incentive spirometry to standard rehabilitation improved peak expiratory flow and reduced dyspnoea in adults with post-COVID-19 condition, supporting its use as a low-cost adjunct for respiratory symptom management.

## LAY ABSTRACT

After recovering from COVID-19, many people continue to experience breathlessness and reduced lung function. This study examined whether adding a simple breathing device called an incentive spirometer to a rehabilitation programme would improve recovery in people with post-COVID-19 condition. Eighty-two adults were randomly divided into 2 groups: 1 group received rehabilitation exercises plus the incentive spirometer, and the other group received rehabilitation alone. Their breathing ability, breathlessness, and physical function were measured before treatment and again after 1 month. Both groups improved. However, participants who used the incentive spirometer showed greater improvements in lung function and breathlessness. Other aspects of recovery, such as mobility and strength, improved similarly in both groups. These results suggest that while rehabilitation is essential for recovery, adding an incentive spirometer may provide additional benefits for breathing in people with post-COVID-19 condition.

In December 2019, COVID-19 emerged in China and rapidly spread worldwide (1). The WHO declared it a pandemic in 2020. To date, more than 779 million confirmed cases (2) and over 7 million deaths (3) have been reported globally.

Common symptoms of the acute infection include fever, dry cough, dyspnoea, anosmia, ageusia, myalgia, fatigue, and general weakness (4–6). These symptoms significantly impact patients’ quality of life physically, psychologically, and socially, placing a considerable burden on healthcare systems (5, 6).

A proportion of individuals experience persistent symptoms following acute infection, a condition referred to as post-COVID-19 condition (PCC). According to the WHO (7), PCC is defined as symptoms that persist or newly appear 3 months after the infection, last at least 2 months, and cannot be explained by an alternative diagnosis. Similarly, the National Academies of Sciences, Engineering and Medicine (NASEM) describe PCC as infection-associated chronic conditions occurring after SARS-CoV-2 infection, highlighting its multisystemic and heterogeneous nature (8). More than 200 symptoms have been reported, with dyspnoea, fatigue, and myalgia among the most prevalent (4–6). Due to methodological heterogeneity across studies, prevalence estimates vary widely, ranging from 6% to over 50%, depending on disease severity, region, follow-up duration, and study design (6, 7, 9–11).

Beyond the clinical burden, PCC has important socioeconomic implications. Increased healthcare utilization and associated expenditures have been reported following COVID-19 infection (12). Furthermore, indirect health consequences, including reduced quality-adjusted life years and substantial economic costs, have been documented (13). These findings underscore the ongoing public health importance of addressing long-term sequelae of COVID-19.

Several mechanisms have been proposed to explain PCC, including persistent inflammation, immune dysregulation, endothelial dysfunction, autoimmunity, and residual organ damage (9, 14, 15). Pulmonary sequelae are particularly common. Persistent interstitial abnormalities, impaired diffusion capacity, restrictive ventilatory patterns, and reduced respiratory muscle performance have been reported months after infection (16–22). These impairments contribute to dyspnoea, reduced exercise tolerance, and diminished quality of life.

Current rehabilitation recommendations emphasize multidisciplinary management, with physiotherapy playing a central role (23). Pulmonary rehabilitation and structured exercise interventions have demonstrated improvements in respiratory function, dyspnoea, functional capacity, fatigue, and quality of life in patients recovering from COVID-19 (24–27).

The incentive spirometer is a low-cost, portable device commonly used to promote sustained maximal inspiration (28–30). It increases lung volumes, facilitates alveolar recruitment, improves ventilation, and supports secretion clearance (28–30). Although an incentive spirometer is widely used in postoperative and chronic pulmonary conditions, high-quality randomized controlled evidence evaluating its clinical effectiveness in patients with PCC remains scarce (31–33). To address this gap, the present randomized controlled trial aimed to assess the clinical effects of incentive spirometer training in patients with PCC undergoing inpatient rehabilitation.

## METHODS

### Study design, randomization, and blinding

This study was a single-centre, parallel-design randomized controlled trial (RCT). Participants were randomly allocated in a 1:1 ratio to receive either incentive spirometer plus standard rehabilitation exercises (experimental group) or standard rehabilitation exercises only (control group).

Randomization was performed using a simple random sequence generated in Microsoft Excel (Microsoft Corp, Redmond, WA, USA). No stratification or block randomization was applied, as the sample size was relatively small and participant characteristics were expected to be homogeneous. Allocation concealment was ensured using sealed, opaque envelopes prepared by a researcher not involved in recruitment or outcome assessment.

Blinding of participants was not feasible, as those in the intervention group were provided with an incentive spirometer. Therapist blinding was also not possible, as the therapist supervised daily physiotherapy sessions. Data collection occurred from 1 January 2023 to 30 September 2023. A pilot study with 10 participants was conducted to evaluate feasibility and study procedures. Participants enrolled in the pilot phase were not included in the main randomized trial and were excluded from the final analysis.

### Participants

The study sample comprised adults previously hospitalized due to acute COVID-19 infection and subsequently diagnosed with PCC. Patients were referred from hospitals across all provinces of the country, for inpatient rehabilitation in the post-COVID ward at the Eden Rehabilitation Centre in Larnaca, Cyprus. All participants were clinically stable at the time of admission and required rehabilitation for persistent post-COVID-19 symptoms.

### Eligibility criteria

*Inclusion criteria.* Adults previously hospitalized due to COVID-19 infection and subsequently diagnosed with PCC, required rehabilitation, haemodynamically stable (conscious, with normal or medically managed heart rate, respiratory rate, and blood pressure), negative rapid COVID-19 test, and able to provided written informed consent.

*Exclusion criteria.* Individuals under 18 years of age, those with medical conditions contraindicating exercise (such as unstable cardiac disease), significant cognitive or psychiatric disorders impairing the ability to follow instructions or provide consent, or unwillingness or inability to participate in the intervention.

### Sampling method

Participants were recruited using consecutive sampling, whereby all individuals meeting the inclusion criteria were considered eligible until the required sample size was achieved. Prior to participation, detailed information was provided regarding the treatment protocol, exercise procedures, safety measures and potential adverse effects.

### Sample size

Sample size calculation was based on 1 of the primary outcomes, peak expiratory flow (PEF, L/min). Based on Kusumawardani et al. (33), who reported mean PEF values of 162 ± 94.34 L/min in the intervention group and 112 ± 71.18 L/min in the control group, a between-group difference of 50 L/min was considered clinically meaningful. The pooled standard deviation derived from these values was approximately 84 L/min. Assuming a two-sided significance level of α = 0.05 and 80% power, a minimum of 23 participants per group (46 total) was required to detect this difference. The sample size calculation was performed using R software (version 4.5.1; https://posit.co/download/rstudio-desktop/; R Foundation for Statistical Computing, Vienna, Austria). To account for potential dropouts and to enhance statistical robustness, 82 participants (41 per group) were recruited. With this sample size, the achieved power was approximately 92% to detect a 50 L/min between-group difference in PEF at α = 0.05.

### Intervention

*Setting and context.* All participants were inpatients admitted to the post-COVID ward at Eden Rehabilitation Centre, Larnaca, Cyprus, receiving multidisciplinary rehabilitation. The ward admitted patients from all provinces of Cyprus. The rehabilitation programme was hospital-based rather than home-based.

*Procedure and monitoring.* Upon admission, patients were screened according to inclusion/exclusion criteria and provided written informed consent. Baseline assessments were conducted on admission, and follow-up measurements were performed 4 weeks later. Before each exercise session, participants’ vital signs (blood pressure, heart rate, oxygen saturation) were recorded to ensure safety. Any adverse events or complaints during or after sessions were documented and reported to the attending clinician or ward nurse. Rehabilitation exercises for both intervention and control groups were administered by an experienced physiotherapist. All participants continued prescribed medications throughout the study.

*Incentive spirometer exercise (experimental group).* Participants in the experimental group performed incentive spirometer exercises in addition to standard rehabilitation. Each participant received an individual flow-based, 3-ball incentive spirometer (Respiprogram®, GIMA SpA, Milan, Italy), and was provided with verbal and written instructions on correct use, along with supervised practice to ensure proper technique. Exercises were performed in a seated position with the device held horizontally at mouth level. Participants were instructed to exhale fully, seal their lips around the mouthpiece, and inhale slowly and deeply to elevate the indicator balls. The breath was held for 2–3 s at maximum inhalation before exhaling (28–30). The exercise protocol consisted of 10 inhalations per session, with 3 sessions, 3 times daily, 6 days per week, for 4 weeks. The intensity and progression of the exercises were individualized according to each participant’s tolerance, with adherence monitored daily by the supervising physiotherapist using individual patient notes.

Dysfunctional breathing patterns were not specifically assessed or corrected prior to training. The incentive spirometer exercises inherently promote slow, deep inhalation and sustained maximal inspiration, supporting appropriate breathing mechanics without additional interventions. Introducing other techniques, such as diaphragmatic breathing exercises, was avoided to ensure that outcomes reflected the effects of the incentive spirometer alone.

*Standard rehabilitation exercises (both groups).* Both the intervention and control groups participated in standard rehabilitation exercises supervised by an experienced physiotherapist. The exercise protocol, based on international guidelines and previous studies in adults with PCC (34–37), included range-of- motion and strengthening exercises for the upper and lower limbs, core control and strengthening, balance exercises, walking with or without walking aids, and assisted or resisted cycling and treadmill walking. Each exercise session lasted 40 min, 6 days per week, for 4 weeks. Exercises were tailored to participants’ functional abilities and progressed based on daily tolerance, as documented in therapist notes, ensuring individualized rehabilitation while maintaining safety.

*Assessment tools and outcome measures.* All outcomes were assessed by a single assessor chosen for validity, reliability, and clinical feasibility. Pulmonary function was measured using peak expiratory flow with a peak flow meter. Participants, seated upright, inhaled deeply and exhaled forcefully into the mouthpiece 3 times, with the highest value recorded. A new disposable mouthpiece was used for each participant to prevent contamination (38). Dyspnoea severity was evaluated using the original Medical Research Council (MRC) Dyspnoea Scale, a 5-point scale from 1 (no dyspnoea) to 5 (severe dyspnoea), where patients selected the statement that best described their symptoms (39). Quality of life was assessed using the EQ-5D-5L questionnaire. For statistical analysis, only the EQ Visual Analogue Scale (EQ-VAS) was used, in which participants rated their overall health status on a scale from 0 (worst imaginable health) to 100 (best imaginable health) at the time of assessment (40). Functional independence was measured with the Barthel Index, assessing 10 activities of daily living, with higher scores indicating greater independence (41). Cardiopulmonary fitness was assessed using the Six-Minute Walk Test (6MWT), which recorded the distance walked in 6 min with rest and walking aids allowed (42). Mobility was evaluated using the Timed Up and Go (TUG) test, timing participants as they stood from a chair, walked 3 m, turned, returned, and sat, with the fastest of 3 trials recorded (43). Lower limb strength was measured using the 30-Second Sit-to-Stand Test (30STS), counting the number of full stands from a chair in 30 s (44). Finally, upper limb strength was measured using a hand-held dynamometer, with the highest value from 3 trials recorded (45). Adherence to all exercises, including incentive spirometer sessions, was monitored daily by the supervising physiotherapist, who recorded attendance, session completion, and any deviations or issues in individual patient notes.

For the 6MWT and the 30STS, raw values (distance in metres and number of repetitions, respectively) were used for analysis. Percentage predicted values were not calculated, and lower limit of normal (LLN) thresholds were not applied. Changes were evaluated based on within- and between-group differences, with adjustment for relevant covariates including gender, marital status, vaccination status, COVID-19 severity, and intubation.

Detailed information on participants’ in-hospital treatment, including duration of mechanical ventilation and use of medications such as corticosteroids, was not available; only clinically relevant data on admission to the rehabilitation centre were recorded.

*Participant’s symptoms at baseline.* At baseline, participants in both groups reported a range of post-COVID-19 symptoms affecting exercise tolerance. In the incentive spirometer group (*n* = 42), 14 patients experienced fatigue, 31 reported muscle weakness, 29 reported dyspnoea, 23 reported reduced endurance, 6 had cough, and 9 reported phlegm production. In the control group (*n* = 42), 15 patients reported fatigue, 33 had muscle weakness, 28 experienced dyspnoea, 21 reported reduced endurance, 4 had cough, and 9 reported phlegm. Post-exertional malaise was not systematically observed during supervised rehabilitation sessions, and exercise progression was individualized based on each participant’s tolerance.

### Statistical analysis

All analyses were conducted according to the intention-to-treat principle using R Statistical Software (version 4.5.1). All randomized participants completed baseline and 4-week follow-up assessments; therefore, no missing data or imputation procedures were required. As no dropouts or protocol deviations occurred, the intention-to-treat and per-protocol populations were identical.

Data distribution was assessed using the Shapiro–Wilk test. Continuous variables are presented as means and standard deviations for normally distributed data, and medians with interquartile ranges for non-normally distributed variables. Categorical variables are presented as frequencies and percentages. A two-sided *p*-value < 0.05 was considered statistically significant.

Baseline characteristics and baseline outcome measures were compared between groups to confirm successful randomization. As no statistically significant baseline differences were observed, between-group comparisons at follow-up were performed using change scores (post-intervention minus baseline). Welch’s *t*-test or the Mann–Whitney *U* test was applied as appropriate based on distribution. Within-group changes were assessed using paired *t*-tests or Wilcoxon signed-rank tests. Categorical variables were compared using the χ^2^ test or Fisher’s exact test, as appropriate.

Mean between-group differences were reported with 95% confidence intervals. The potential influence of covariates (gender, marital status, vaccination status, COVID-19 severity, and intubation history) was explored in secondary analyses. To account for multiple comparisons across the eight outcome measures, the Benjamini–Hochberg false discovery rate (FDR) correction was applied where appropriate.

## RESULTS

**Fig. 1:**
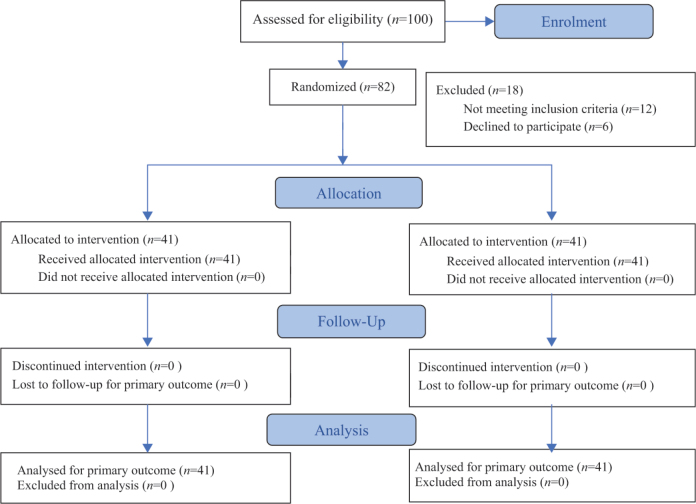
Consort 2025 flow diagram.

A total of 100 patients with PCC were screened for eligibility. Eighty-two participants met the inclusion criteria and were randomly assigned to the experimental group (*n* = 41) or the control group (*n* = 41) (Fig. 1). Baseline demographic characteristics and outcome measures were comparable between groups (Tables I and II).

**Table I:** Baseline characteristics

Characteristics	Control group	Experimental group	*p*-value
Age (years)	70.98 (12.76)	69.93 (10.48)	0.685
Gender			
Male	20 (48.78%)	20 (48.78%)	1.000
Female	21 (51.22%)	21 (51.22%)	
Occupation			
Retired	30 (73.17%)	28 (68.29%)	0.379
Other*	11 (26.83%)	13 (31.71%)	
Covid severity			
Moderate	23 (56.10%)	24 (58.54%)	0.823
Severe	18 (43.90%)	17 (41.46%)	
Family status			
Married	31 (75.61%)	30 (73.17%)	0.800
Other	10 (24.39%)	11 (26.83%)	
Intubation			
Yes	18 (43.90%)	17 (41.46%)	0.823
No	23 (56.10%)	24 (58.54%)	
Vaccination			
Yes	15 (36.59%)	16 (39.02%)	0.820
No	26 (63.41%)	25 (60.98%)	
Comorbidities			
Hypertension	28 (68.29%)	29 (70.73%)	0.810
Diabetes	20 (48.78%)	18 (43.90%)	0.657
High cholesterol	15 (36.59%)	13 (31.71%)	0.641
Respiratory disease	11 (26.83%)	13 (31.71%)	0.627
Cardiovascular disease	13 (31.71%)	12 (29.27%)	0.810
Neurological disease	9 (21.95%)	8 (19.51%)	0.785
Cancer	8 (19.51%)	8 (19.51%)	1.000
Renal disease	8 (19.51%)	6 (14.63%)	0.557
Baseline symptoms†			
Fatigue	15 (36.59%)	14 (34.15%)	
Muscle weakness	33 (80.49%)	31 (75.61%)	
Dyspnoea	28 (68.29%)	29 (70.73%)	
Reduce endurance	21 (51.22%)	23 (56.10%)	
Cough	4 (9.76%)	6 (14.63%)	
Phlegm production	9 (21.95%)	9 (21.95%)	

Values are presented as mean (SD) for continue data and number (percentage) for categorical data.

* Other occupations include salesman, civil engineer, housewife, real estate agent, cleaner, electrician, priest, seamstress, unemployed, accountant, taxi driver, pharmacist, and pastry chef.†Symptom counts reflect number of participants reporting each symptom at baseline. Symptom severity was assessed using a standardized checklist based on self-reported symptom burden and WHO Clinical management of COVID-19: living guideline: June 2025(7).

**Table II:** Effect of the study interventions on clinical parameters with exploratory covariate checks

Clinical parameter	Control baseline (mean (SD)/median [IQR])	Experimental baseline (mean (SD)/median [IQR])	Baseline *p*-value	Control 1 month (mean (SD)/median [IQR])	Experimental 1 month (mean (SD)/median [IQR])	Between-group difference (95% CI)	1-month *p*-value	Covariate exploratory findings (1 month)
PEF (L/min)	133.90 (94.76)	110.73 (76.60)	0.434	245.61 (170.94)	311.46 (105.61)	65.85 (4.35, 127.35)	0.007	No covariates significantly affected PEF (*p*_adj > 0.39 for all)
MRC dyspnoea (1–5)	4.22 (0.85)	4.51 (0.75)	0.090	3.42 (1.14)	2.34 (0.99)	–1.08 (–1.54, –0.62)	< 0.001	No covariates significantly affected MRC (*p*_adj > 0.39 for all)
EQ-5D-5L (VAS 0–100)	50.73 (19.19)	50.24 (22.36)	0.840	74.27 (22.40)	74.39 (25.62)	0.12 (–12.30, 12.52)	0.839	No covariates significantly affected EQ-5D-5L (*p*_adj > 0.39 for all)
Barthel Index	35.24 [20–45]	43.78 [25–60]	0.123	62.32 (28.51)	71.10 (29.36)	8.78 (–6.50, 24.00)	0.186	No covariates significantly affected Barthel (*p*_adj > 0.49)
30STS	1.12 (1.65)	1.44 (2.18)	0.975	4.07 (3.69)	5.12 (4.50)	1.05 (–1.20, 3.30)	0.492	No covariates significantly affected 30sSTS (*p*_adj > 0.56)
6MWT (m)	4.27 (18.22)	7.68 (20.38)	0.328	116.71 (159.47)	87.07 (134.62)	–29.64 (–95.10, 35.80)	0.667	No covariates significantly affected 6MWT (*p*_adj > 0.44)
TUG (s)	20.50 (6.70)	21.30 (7.58)	0.812	14.96 (5.98)	15.47 (4.94)	–1.57 (–5.41, 2.26)	0.414	No covariates significantly affected TUAG (*p*_adj > 0.50)
Hand Grip (kg)	14.03 (4.53)	14.50 [11.10–18.90]	0.234	17.03 (4.95)	17.40 [15.00–22.30]	0.37 (–1.25, 1.99)	0.430	Gender affected handgrip in experimental group (*p*_adj=0.035), no other covariates were significant

Values are presented as mean (SD) for normally distributed data and median [IQR] for skewed data. Baseline *p*-values reflect unadjusted group differences. Change scores represent within-group improvements from baseline to 1 month. Between-group differences at 1 month are presented as raw mean differences and adjusted estimates with 95% confidence intervals from exploratory covariate analyses. PEF, MRC, and EQ-5D-5L (VAS) are the primary outcomes. Clinically meaningful improvements were observed for PEF (+65.85 L/min) and MRC (–1.08 points), exceeding minimal detectable changes reported in similar populations. MRC: Medical Research Council Dyspnoea Scale; PEF: Peak expiratory flow; 30STS: 30-second Sit-to-Stand Test; 6MWT: Six-Minute Walk Test; TUG: Timed Up and Go; EQ-5D-5L (VAS 0-100): EuroQoL 5D 5L Visual analogue scale 0–100.

### Primary outcomes

Peak expiratory flow (PEF) improved in both groups from baseline to 1 month. In the experimental group, mean PEF increased from 110.73 ± 76.60 L/min at baseline to 311.46 ± 105.61 L/min at follow-up. In the control group, PEF increased from 133.90 ± 94.76 L/min to 245.61 ± 170.94 L/min. Between-group comparison at one month indicated a statistically significant difference favouring the experimental group (difference = 65.85 L/min; 95% CI: 4.35 to 127.35; *p* = 0.007). This magnitude of change is consistent with a clinically meaningful improvement in expiratory flow for patients with chronic lung diseases.

Dyspnoea, measured using the MRC scale, improved from 4.51 ± 0.75 at baseline to 2.34 ± 0.99 at 1 month in the experimental group. In the control group, dyspnoea improved from 4.22 ± 0.85 to 3.42 ± 1.14. Between-group comparison at 1 month showed a significant difference favouring the experimental group (difference = –1.08; 95% CI: –1.54 to –0.62; *p* < 0.001), representing a clinically meaningful reduction in dyspnoea severity.

Quality of life, assessed with the EQ-5D-5L (VAS score), improved in both groups from baseline to 1 month. However, the between-group difference at follow-up was not statistically significant (experimental: 74.39 ± 25.62 vs control: 74.27 ± 22.40; *p* = 0.839).

### Secondary outcomes

No statistically significant between-group differences were observed at 1 month for the Barthel Index, 30STS, 6MWT, TUG, or handgrip strength. Both groups demonstrated improvements over time from baseline to follow-up. Detailed baseline, follow-up, and between-group differences with 95% confidence intervals are provided in Table II.

### Effect of demographic covariates

Exploratory analyses adjusting for gender, marital status, vaccination status, COVID-19 severity, and intubation demonstrated that only handgrip strength differed by gender in the experimental group on admission (*p*_adj = 0.024) and at 1 month (*p*_adj = 0.035). No other outcome measures were significantly influenced by these covariates after Benjamini*–*Hochberg false discovery rate correction (see Table II).

### Adherence and safety

All participants adhered fully to their assigned rehabilitation programmes, as documented by daily therapist notes. No adverse events or complications related to the intervention were reported in either group.

## DISCUSSION

The aim of this study was to evaluate the effects of incentive spirometer use on the recovery of adults with post-COVID-19 condition. The findings indicate that adding an incentive spirometer to a standard rehabilitation programme led to statistically and clinically significant improvements in peak expiratory flow and dyspnoea, as measured by the MRC scale, compared with the control group receiving rehabilitation alone.

### Primary outcomes

PEF improved substantially in the experimental group, with a mean PEF increase of 200.73 L/min from baseline to 1 month, compared with 111.71 L/min in the control group (between-group difference = 65.85 L/min; 95% CI: 4.35 to 127.35; *p* = 0.007). Although a formal MCID for PEF in post-COVID populations has not been established, changes of approximately 40–70 L/min have been linked to meaningful improvements in ventilatory capacity, symptom burden, and exercise tolerance in chronic respiratory disease and pulmonary rehabilitation studies (46). Similarly, the MRC dyspnoea scale showed a significant reduction in the experimental group relative to the control group (difference = –1.08; 95% CI: –1.54 to –0.62; *p* < 0.001). The reduction in dyspnoea by ~1 point on the MRC scale is considered clinically meaningful in patients with chronic respiratory disease (47). These improvements are functionally meaningful, as enhanced PEF and reduced dyspnoea can directly translate into better exercise tolerance, increased ability to perform activities of daily living, and improved overall functional capacity in PCC patients.

Quality of life, while improved in both groups, did not differ significantly between the experimental and control groups. This suggests that the addition of an incentive spirometer provided specific benefits to respiratory function and breathlessness, rather than broader psychosocial outcomes over the short follow-up period.

The observed improvements in PEF and dyspnoea are consistent with the physiological effects of incentive spirometer use, which promotes alveolar recruitment, enhances inspiratory capacity, and supports optimal ventilatory mechanics. Increased expiratory flow can improve airway clearance, reduce ventilatory effort, and support functional activities, while reduction in dyspnoea may increase participation in daily activities and physical exercise, enhancing overall recovery. Visual feedback may further enhance adherence and effort, promoting more effective ventilatory patterns (48).

### Secondary outcomes

Other outcomes, including functional independence, mobility, and strength measures, improved in both groups but did not differ significantly. This likely reflects the robust effect of the comprehensive rehabilitation programme provided to all participants, which included endurance, strength, and balance exercises. The observed within-group improvements across multiple domains likely reflect the broad benefits of structured rehabilitation, whereas the additive effect of the incentive spirometer was primarily observed in outcomes directly related to respiratory mechanics.

### Comparison with previous studies

These findings align with previous research demonstrating the efficacy of incentive spirometer in PCC and other chronic respiratory conditions. Loganathan et al. (32) reported that incentive spirometer use significantly improved pulmonary function parameters, including FVC, FEV_1_, and DLCO, compared to pharmacotherapy alone. Kusumawardani et al. (33) showed improvements in PEF, while Abo Elyazed et al. (31) demonstrated reductions in MRC dyspnoea scores following incentive spirometer interventions.

Similarly, studies by Tengker et al. (49) and Gudivada et al. (50) reported enhanced cough and ventilatory function as well as reductions in anxiety and depression scores in patients undergoing incentive spirometer training. Some studies, however, such as Suharti et al. (51), reported no significant between-group differences in functional capacity, highlighting the potential impact of comprehensive rehabilitation on all participants. Our findings reflect this mixed evidence that the beneficial effects of incentive spirometer are most pronounced in measures closely related to ventilatory function, whereas general improvements in mobility, strength, and quality of life are largely attributable to the rehabilitation programme.

### Mechanistic and functional implications

Importantly, improvements in PEF and dyspnoea observed in the experimental group are likely to have meaningful functional implications. Higher expiratory flow and reduced breathlessness can facilitate greater participation in physical activity, improve endurance, and enhance the ability to perform activities of daily living. These respiratory gains may also contribute indirectly to better quality of life by reducing fatigue, increasing confidence in mobility, and supporting engagement in social and occupational activities. Thus, the addition of an incentive spirometer to standard rehabilitation not only targets pulmonary mechanics but may also amplify overall functional recovery in PCC patients.

Exercise included in the rehabilitation programme also contributes to improvements in muscle mass, strength, cardiovascular fitness, and psychological well-being, explaining why both groups showed within-group gains across multiple outcome measures. Pulmonary rehabilitation has been shown to improve exercise capacity, reduce fatigue, and enhance both physical and mental health in PCC populations (24, 27, 52–53).

### Ethical considerations

All participants received standard rehabilitation to ensure ethical management of patients with persistent PCC symptoms, preventing deprivation of beneficial therapy while allowing assessment of the additive effects of an incentive spirometer.

### Limitations

The findings of the present study should be interpreted in light of several limitations. First, the study was conducted at a single centre, which may limit the generalizability of the results. Second, the follow-up period was relatively short (1 month), restricting conclusions concerning the sustainability of observed improvements. Third, measures of inspiratory muscle strength were not included, limiting insight into the specific physiological mechanisms underlying the observed benefits. We have prioritized outcome measures that were validated, reliable, and quick to administer to minimize participant burden. Fourth, due to the nature of the intervention, blinding of participants and treating physiotherapist was not feasible, which may introduce performance bias. To mitigate detection bias, all outcome assessments were conducted by an independent physiotherapist who was blinded to group allocation and unaware of the study objectives. Furthermore, the sample size was modest, which may limit the statistical power to detect smaller between-group differences for some secondary outcomes. Finally, the absence of detailed hospitalization data, including mechanical ventilation duration and specific treatments, is a limitation, as these factors may have influenced baseline functional status and recovery trajectories.

### Future studies

Further research with multi-centre designs, longer follow-up periods, and inclusion of inspiratory muscle strength assessments is warranted to confirm and extend these findings. Evaluating cost-effectiveness and comparing incentive spirometer-integrated rehabilitation with other interventions will provide additional insight into optimal management strategies for patients with PCC.

### Implications for practice

The findings of this study reinforce the importance of structured rehabilitation in managing individuals with PCC, demonstrating significant improvements in quality of life, functional independence, cardiopulmonary function, mobility, and muscular strength across both the intervention and control groups. Although the addition of an incentive spirometer led to statistically significant improvements in pulmonary function and dyspnoea, other outcome measures did not differ between groups, suggesting that structured rehabilitation alone is highly effective.

The incentive spirometer remains clinically relevant as a low-cost, non-invasive, and easy-to-use adjunct that provides visual feedback and may enhance patient engagement and adherence to respiratory exercises. For patients with persistent respiratory symptoms, such as dyspnoea or reduced pulmonary function, incentive spirometry offers a targeted method to promote lung expansion and improve inspiratory muscle performance.

However, it is important to acknowledge that other breathing interventions, including diaphragmatic breathing exercises, inspiratory muscle training devices, and general respiratory exercises, may also yield similar benefits. An incentive spirometer should therefore be considered as part of a multimodal rehabilitation programme rather than as a standalone or superior intervention. Rehabilitation professionals should tailor the choice of respiratory exercises to individual patient needs, resources, and feasibility, ensuring that an incentive spirometer complements rather than replaces comprehensive rehabilitation strategies.

In conclusion, the addition of an incentive spirometer to standard rehabilitation in adults with PCC significantly improved PEF and dyspnoea, indicating a clinically meaningful enhancement in respiratory function. Our findings suggest that the incentive spirometer is a safe, low-cost, and effective adjunct to standard pulmonary rehabilitation for this patient population.
